# Systematic analysis of the lysine acetylome in *Fusarium graminearum*

**DOI:** 10.1186/s12864-016-3361-3

**Published:** 2016-12-13

**Authors:** Shanyue Zhou, Qianqian Yang, Changfa Yin, Lin Liu, Wenxing Liang

**Affiliations:** 1 College of Agronomy and Plant Protection, The Key Lab of Integrated Crop Pests Management of Shandong Province, Qingdao Agricultural University, No. 700 Changcheng Road, Chengyang, Qingdao, Shandong 266109 China; 2 College of Plant Protection, China Agricultural University, Beijing, 100193 China; 3 College of Life Sciences, Shandong Province Key Laboratory of Applied Mycology, Qingdao Agricultural University, Qingdao, 266109 China

**Keywords:** Lysine acetylation, Post-translational modification, Acetylproteome, *Fusarium graminearum*

## Abstract

**Background:**

Lysine acetylation in proteins is a ubiquitous and conserved post-translational modification, playing a critical regulatory role in almost every aspect of living cells. Although known for many years, its function remains elusive in *Fusarium graminearum*, one of the most important necrotrophic plant pathogens with huge economic impact.

**Results:**

By the combination of affinity enrichment and high-resolution LC-MS/MS analysis, large-scale lysine acetylome analysis was performed. In total, 577 lysine acetylation sites matched to 364 different proteins were identified. Bioinformatics analysis of the acetylome showed that the acetylated proteins are involved in a wide range of cellular functions and exhibit diverse subcellular localizations. Remarkably, 10 proteins involved in the virulence or DON (deoxynivalenol) biosynthesis were found to be acetylated, including 4 transcription factors, 4 protein kinases and 2 phosphatases. Protein-protein interaction network analysis revealed that acetylated protein complexes are involved in diversified interactions.

**Conclusions:**

This work provides the first comprehensive survey of a possible lysine acetylome in *F. graminearum* and reveals previously unappreciated roles of lysine acetylation in the regulation of diverse biological processes. This work provides a resource for functional analysis of acetylated proteins in filamentous fungi.

**Electronic supplementary material:**

The online version of this article (doi:10.1186/s12864-016-3361-3) contains supplementary material, which is available to authorized users.

## Background

Acetylation of lysine residues in proteins is a dynamic and reversible post-translational modification (PTM) occurring ubiquitously in living cells in both prokaryotes and eukaryotes [1–4]. Lysine acetylation is catalyzed by lysine acetyltrasferases (KATs) and reversed by lysine deacetylases (KDACs) [5]. Through this reversible process the acetylation status of proteins is dynamically balanced for proper cellular regulation [6, 7]. Since the first discovery of lysine acetylation in histones, the acetylation of protein was extensively studied [8]. Moreover, up to now, many non-histone proteins have been identified to be lysine acetylated as well. The well-characterized acetylated non-histone proteins mainly include metabolic enzymes, transcription factors, hormone receptors, signal transducers, chaperones and proteins of the cytoskeleton [9, 10]. The studies of the non-histone targets have greatly expanded our understanding of this PTM in cellular processes.

In recent years, with the advance of mass spectrometry (MS) and high affinity purification of acetylated peptides, acetylproteome studies have been successfully performed in some prokaryotic microorganisms, such as *Escherichia coli* [11], *Salmonella enterica* [12], *Bacillus subtilis* [13], *Geobacillus kaustophilus* [14], *Erwinia amylovora* [15], *Thermus thermophilus* [16] and *Saccharopolyspora erythraea* [4]. However, compared with bacteria, the acetylome of fungi is poorly studied. Until now, acetylproteome study has only been reported in the budding yeast, *Saccharomyces cerevisiae* [5], latestly reported in Oomycete, *Phytophthora sojae* [17] and in filamentous fungi, *Botrytis cinerea* [18].

*Fusarium graminearum* is the causal pathogen of *Fusarium* head blight (FHB) on wheat and barley. Infection of *F. graminearum* not only results in enormous grain yield loss, but also contaminates grains with mycotoxins which exhibit toxicity to humans and other mammals [19, 20]. Genome analysis revealed that there are a large number of acetyltranferase and deacetylase orthologs in the genome of *F. Graminearum* [21]. Functional studies on some deacetylase genes in plant pathogens, for example FTL1 in *F. graminearum* [22], Tig1 in *Magnaporthe oryzae* [23], and FfHDA1 and FfHDA2 in *Fusarium fujikuroi* [24], showed that these deacetylase genes play important regulatory roles in fungal pathogenesis. The presence of acetyltranferase and deacetylase orthologs suggests that acetylation of proteins may play a critical role in the pathogenesis of *F. graminearum*. To test this hypothesis, we carried out a proteomics study of lysine acetylation in *F. graminearum* PH-1. By the combination of high affinity enrichment and high-resolution LC-MS/MS analysis, we identified 577 acetylated sites in 364 different proteins controlling various biological processes in this fungus. The acetylated proteins are localized in multiple compartments with diverse functions. Importantly, 10 proteins involved in virulence or DON biosynthesis were found to be acetylated. These results provide a system-wide view of the *F. graminearum* acetylome and an affluent dataset for functional analysis of acetylated proteins in this important plant pathogen.

## Methods

### Fungal strain and culture

The fungus of *F. graminearum* PH-1 was incubated on potato dextrose agar (PDA) at 25 °C. The conidia were induced and harvested following a described protocol [25]. The conidia were incubated in YEPD (0.3% yeast extract, 1% peptone, 2% dextrose) medium at 25 °C with shaking at 200 rpm for 20 h and the hyphae were harvested by filtering with sterile macrocloth.

### Protein extraction and trypsin digestion

Hyphae were grinded into cell powder in liquid nitrogen [26, 27]. The cell power was transferred into 5 ml lysis buffer (8 M urea, 1% Triton-100, 65 mM dithiothreitol (DTT), 0.1% Protease Inhibitor Cocktail IV, 3 μM trichostatin A, 50 mM nicotinamide and 2 mM EDTA), and sonicated three times on ice using a high intensity ultrasonic processor (Scientz, Ningbo, China). The debris was removed by centrifugation at 20,000 × g at 4 °C for 10 min. Finally, the proteins were precipitated with 15% cold TCA for 2 h at −20 °C. After centrifugation at 4 °C for 10 min, the supernatant was discarded. The remaining precipitate was washed with cold acetone for three times and then resolved in buffer (8 M urea, 100 mM (NH_4_)_2_CO_3_, pH 8.0). Protein concentration was determined with 2-D Quant kit (GE Healthcare) according to the manufacturer’s instructions.

The resulting protein solution was reduced with 10 mM DTT for 1 h at 37 °C and alkylated with 20 mM iodoacetamide for 45 min at room temperature in darkness. For trypsin digestion, the protein sample was diluted by adding 100 mM (NH_4_)_2_CO_3_ to urea concentration less than 2 M. Finally, trypsin was added at 1:50 trypsin-to-protein mass ratio for the first digestion overnight and 1:100 trypsin-to-protein mass ratio for the second 4 h-digestion.

### HPLC fractionation and affinity enrichment

The sample was separated into fractions by high pH reverse-phase HPLC using Agilent 300 Extend C18 column (5 μm particles, 4.6 mm ID, 250 mm length). Briefly, peptides were first separated with a gradient of 2 to 60% acetonitrile in 10 mM ammonium bicarbonate (pH 10.0) over 80 min into 80 fractions. Then, the peptides were combined into 6 fractions and dried by vacuum centrifuging.

The purification and enrichment of lysine acetylated peptides was followed [17] and [18]. Briefly, tryptic peptides dissolved in NETN buffer (100 mM NaCl, 1 mM EDTA, 50 mM Tris-HCl, 0.5% NP-40, pH 8.0) were incubated with pre-washed agarose-conjugated anti-acetyllysine antibody beads (Cat. No. 104, PTM Biolabs, Hangzhou, China) at 4 °C overnight with gentle shaking. The beads were washed four times with NETN buffer and twice with ddH_2_O. The bound peptides were eluted from the beads with 0.1% trifluoroacetic acid. The eluted fractions were combined and vacuum-dried. The resulting peptides were cleaned with C18 ZipTips (Millipore, Billerica, MA) according to the manufacturer’s instructions, followed by LC-MS/MS analysis.

### LC-MS/MS analysis

Peptides were dissolved in 0.1% formic acid (FA) and directly loaded onto a reversed-phase pre-column (Acclaim PepMap100 C18 column, 3 μm, 75 μm × 2 mm, 100 Å, Thermo Scientific). Peptide separation was performed using a reversed-phase analytical column (Acclaim PepMap RSLC C18 column, 2 μm, 50 μm × 15 mm, 100 Å, Thermo Scientific). The gradient was composed of an increase from 7 to 20% solvent B (0.1% FA in 98% acetonitrile) for 20 min, 20 to 35% for 8 min and climbing to 80% in 2 min then holding at 80% for the last 5 min, all at a constant flow rate of 300 nl/min on an EASY-nLC 1000 UPLC system. The resulting peptides were subjected to electrospray/nanospray ionization (ESI/NSI). Intact peptides were detected in the Orbitrap at a resolution of 70,000 (m/z 200). Peptides were selected for MS/MS using NCE setting as 33; ion fragments were detected in the Orbitrap at a resolution of 17,500 (m/z 200). A data-dependent procedure that alternated between one MS scan followed by 16 MS/MS scans was applied for the top 16 precursor ions above a threshold ion count of 1.5E^4^ in the MS survey scan with 10.0 s dynamic exclusion. The electrospray voltage applied was 2.0 kV. Automatic gain control was used to prevent overfilling of the ion trap. MS1 spectra were obtained with an AGC target of 3E^6^ ions and a maximum injection time of 50 ms, and MS2 spectra were acquired with an AGC target of 5E^4^ ions and a maximum injection time of 200 ms. For MS scans, the m/z scan range was 350 to 1800. Fixed first mass was set as 100 m/z.

### Database search

The resulting MS/MS data was processed using MaxQuant with integrated Andromeda search engine (v.1.4.1.2). Tandem mass spectra were searched against UniProt *F. graminearum* (27,754 sequences) database concatenated with reverse decoy database. Trypsin was specified as cleavage enzyme allowing up to 4 missing cleavages, 5 modifications per peptide and 5 charges. Mass error was set to 10 ppm for precursor ions and 0.02 Da for fragment ions. Carbamidomethylation on Cys was specified as fixed modification and oxidation on Met, acetylation on lysine and acetylation on protein N-terminal were specified as variable modifications. False discovery rate (FDR) thresholds for peptide and modification site were specified at 1%. Minimum peptide length was set as 7. All the other parameters in MaxQuant were set to default values. The site localization probability was set as 0.75.

### Bioinformatics analyses

Gene Ontology (GO) annotation of acetylome was derived from the UniProt-GOA database (http://www.ebi.ac.uk/GOA/). Firstly, the identified protein ID was converted to UniProt ID, and then mapped to GO ID by protein ID. If some identified proteins were not annotated by UniProt-GOA database, the InterProScan5 was used to annotate protein’s GO function based on protein sequence alignment [28]. The proteins were classified by GO annotation based on three categories: biological process, cellular component and molecular function. The subcellular localization of the protein was predicted with WoLF PSORT, a subcellular localization prediction program (http://wolfpsort.seq.cbrc.jp/). Secondary structures of proteins were predicted by NetSurfP [29]. Motif-x [30] was used to analyze the model of identified sequences constituted with amino acids in specific positions of modified-20-mers (10 amino acids upstream and downstream of the acetylated site) in all protein sequences. Domain descriptions of identified protein were annotated by InterProScan5 based on protein sequence alignment, and the InterPro domain database (http://www.ebi.ac.uk/interpro/) was used. Kyoto Encyclopedia of Genes and Genomes (KEGG) database was used to annotate protein pathway [31]. Functional annotation tool of DAVID bioinformatics resources 6.7 was used to identify GO terms, KEGG pathways and protein domains [28]. A two-tailed Fisher’s exact test was performed to examine the enrichment of the protein-acetylated entries against all proteins. Correction for multiple hypothesis testing was carried out using a previously described method [32]. Any term with a corrected *p* < 0.05 was considered significant. Analysis on physical and functional interaction network of the identified proteins was performed using STRING database [33] and was visualized using Cytoscape (version 3.2.0, www.cytoscape.org).

## Results and discussion

### Identification and analysis of lysine-acetylated proteins in *F. graminearum*

Combination of immune-affinity purification and enrichment and high-resolution LC-MS/MS proteomic method was employed to determine the acetylome of *F. graminearum* PH-1. The distribution of mass errors was near zero and most of them were less than 5 ppm (Additional file 1: Figure S1A) which means the mass accuracy of the MS data fits the requirement. The length of most peptides was distributed between 7 and 25 amino acids (Additional file 1: Figure S1B), which agrees with the property of tryptic peptides and means sample preparation reaches the standard. As such, we identified 1462 peptides, including 913 unmodified peptides and 544 acetylated peptides. The modified peptides, with 577 lysine acetylation sites identified, were matched to 364 different proteins, which account for 2.73% of the total proteins (Additional file 2: Table S1). The ratio of the acetylated proteins to the total proteins in *F. graminearum* is much lower than that in *P. sojae* (6%) [17] and *B. cinerea* (5.82%) [18]. However, the similar significant difference was also found in different bacterial species [3]. The strategy and technologies used to analyze lysine acetylation in *F. graminearum* were the same to be used in *P. sojae*, and *B. cinerea*. So the intrinsic acetylation level of proteins varying markedly between different microbe species maybe a reasonable interpretation to the significant difference between *F. Graminearum* and *P. sojae* or *B. cinerea*.

### Analysis of acetylated lysine sites

**Fig. 1:**
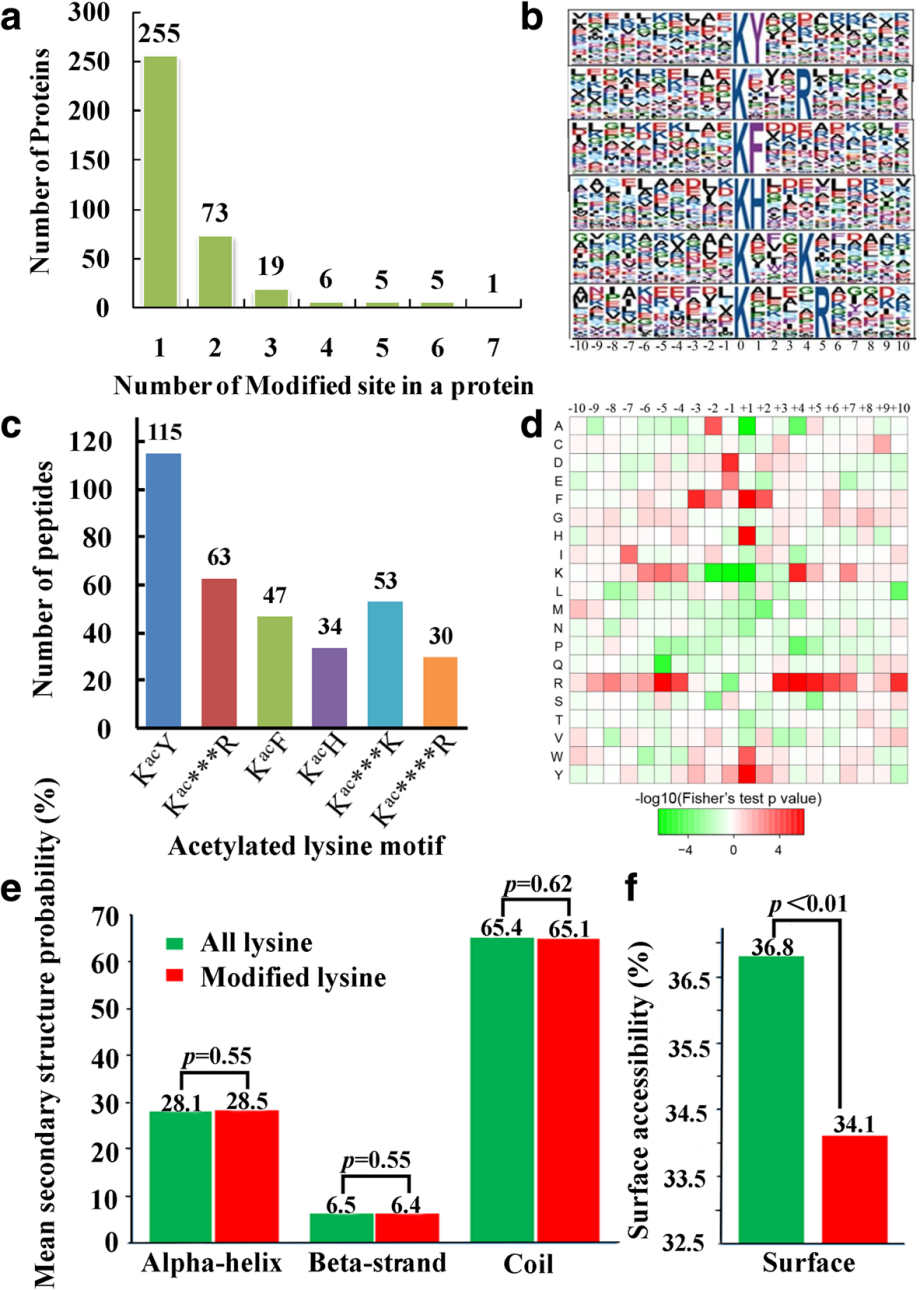
Properties of the acetylated sites. **a** Distribution of acetylated proteins based on their number of acetylation sites. Numbers of proteins with different acetylation sites (1–7) were shown on top of the columns. **b** Acetylation motifs and conservation of acetylation sites. The size of each letter corresponds to the frequency of the amino acid residue in that position. The central K refers to the acetylated lysine. **c** Distribution of identified proteins based on the acetylation motifs. Numbers of peptides with different conserved motifs were shown on top of the columns. **d** Heat map of the amino acid compositions of the acetylation sites showing the frequency of different amino acids around the acetylated lysine. Red indicates high frequency and green means low frequency. **e** Probabilities of lysine acetylation in different protein secondary structures. Different secondary structures (alpha-helix, beta-strand and coil) of acetylated lysine residues identified in this study were compared with those of all lysine on all proteins. **f** Predicted surface accessibility of acetylated sites. Surface accessibility of the acetylated lysine sites was compared with that of all lysine

In order to assess the distribution of acetylation sites in the acetylated proteins of *F. graminearum*, the numbers of identified modification sites per protein were calculated. The results showed that 70% of proteins contained only one acetylation site, and the percentage of proteins with two, three and four or more modification sites were 20, 5 and 5%, respectively (Fig. 1a).

To better understand the acetylation motifs in *F. graminearum*, the context of amino acid sequence surrounding the acetylated lysines was analyzed. Six significantly enriched motifs, namely K^ac^Y, K^ac^***R, K^ac^F, K^ac^H, K^ac^***K and K^ac^****R (Kac indicates the acetylated lysine and * indicates a random amino acid residue), were identified from 530 unique acetylation sites accounting for 91.9% of the modification sites identified (Fig. 1b). These six motifs exhibited different abundances, and motifs K^ac^Y, K^ac^***R and K^ac^***K were particularly conserved as peptides with these motifs account for approximately 68% of all the identified peptides (Fig. 1c). Moreover, the heat map of amino acid compositions surrounding the acetylation sites showed that the frequency of phenylalanine (F), tyrosine (Y) and histidine (H) in position +1 in these motifs is the highest, whereas the frequency of K and R in positions −2 to +1 is the lowest (Fig. 1d). Therefore, proteins with such motifs are preferred substrates of lysine acetyltransferases in *F. graminearum* cells. In addition, most of the motifs found in *F. graminearum* have also been found in other organisms [34–38], indicating the high conservation of lysine acetylation among different species. Except the 6 motifs in *F. graminearum*, another 4 motifs FK^ac^, YK^ac^, LK^ac^ and EK^ac^ were found in *B. cinerea* [18], which maybe contribute to interpret the higher ratio of acetylated proteins in *B. cinerea*. Surprisingly, 15 motifs were found in *P. sojae* [17], among which only 3 ones were found in *F. graminearum* and 6 ones were found in *B. cinerea*. Maybe the difference is closely related to the evolution and variation of different organisms.

To elucidate the relationship between lysine acetylation and protein structure, the local secondary structure of acetylated proteins was investigated (Fig. 1e). Acetylation sites were more frequently located at coil region, compared with alpha-helix and beta-strand, suggesting that the acetylation might prefer the disordered structure in *F. graminearum* proteins. Based on the similarity of distribution pattern between acetylated lysine and all lysine (*p* = 0.55, 0.55 and 0.62 for α-helix, β-strand and coil, respectively), it seems there is no tendency of acetylation in *F. graminearum* proteins. Surface accessibility of the acetylated lysine sites was also evaluated. The results showed that, compared with 36.8% of all lysine residues, 34.1% of the acetylated lysine sites were exposed to protein surface (*p* < 0.01) (Fig. 1f). Therefore, surface accessibility of proteins is slightly affected by lysine acetylation.

### Functional annotation and cellular localization of acetylated proteins in *F. graminearum*

**Fig. 2:**
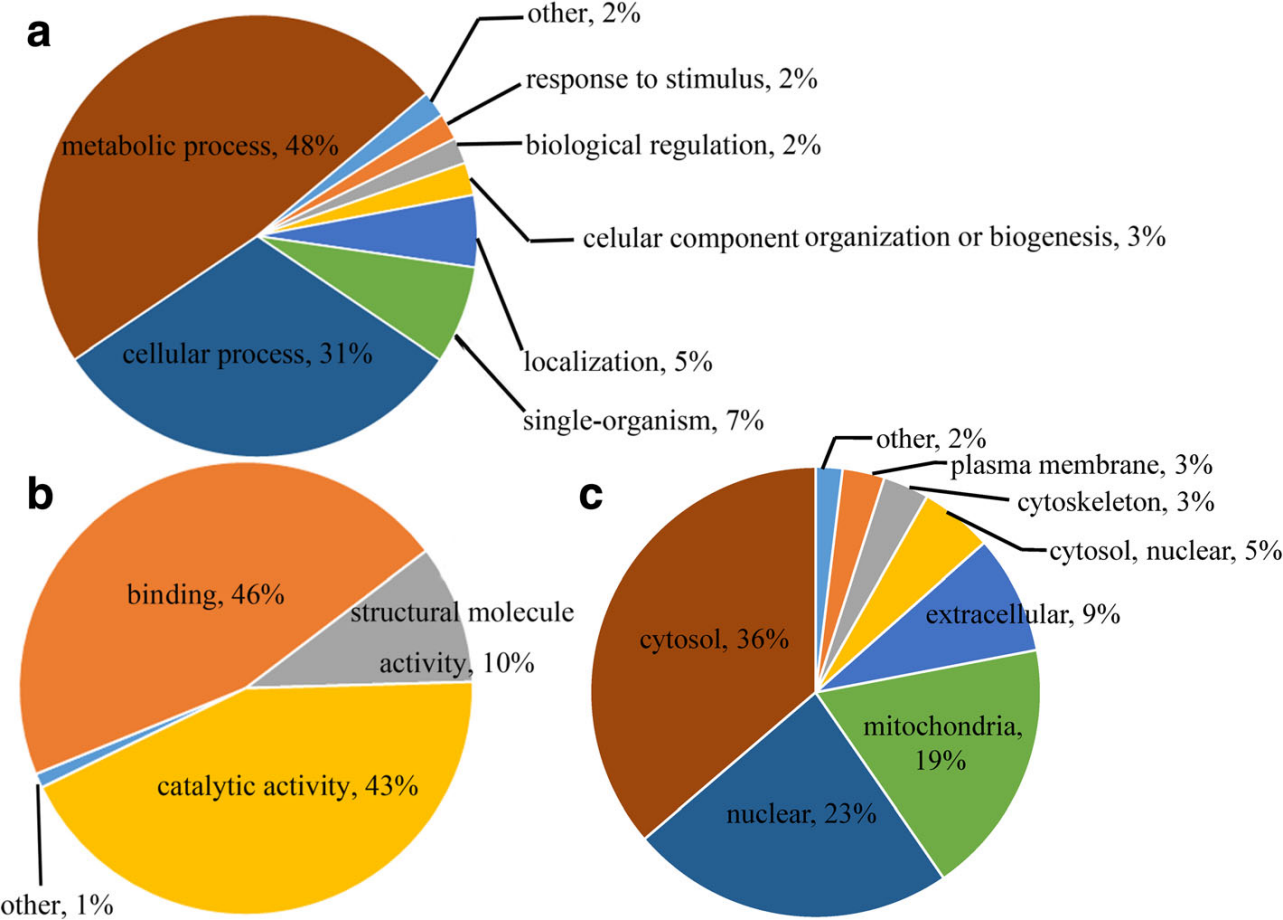
Functional classification of acetylated proteins in *F. graminearum*. **a** Classification of the acetylated proteins based on biological process. **b** Classification of the acetylated proteins based on molecular function. **c** Subcellular localization of the acetylated proteins. The acetylated proteins were classified by GO annotation based on biological process and molecular function. The subcellular localization of acetylated proteins was predicted with WoLF PSORT. The percentage of acetylated proteins in each category was shown in individual pie charts

To better understand the lysine acetylome in *F. graminearum*, we annotated and classified the identified proteins according to biological process and molecular function. Consistent with previous acetylome reports of other microbes, the GO analysis of the acetylome showed that the acetylated proteins have wide ranges of biological processes and molecular functions in *F. graminearum*. The largest group of acetylated proteins consists of enzymes that are associated with metabolism (48%) in the biological process classification (Fig. 2a). Most acetylated proteins were found to be related to binding (46%) and catalytic activity (43%) in the molecular function classification (Fig. 2b). Subcellular localization of the acetylated proteins was also analyzed and it showed that most of the modified proteins are located in cytosol (36%), nucleus (23%) and mitochondria (19%) (Fig. 2c). Collectively, these results indicate that the acetylated proteins, with diverse functions, are widely distributed in *F. graminearum* cells.

### Functional enrichment analysis of acetylated proteins

**Fig. 3:**
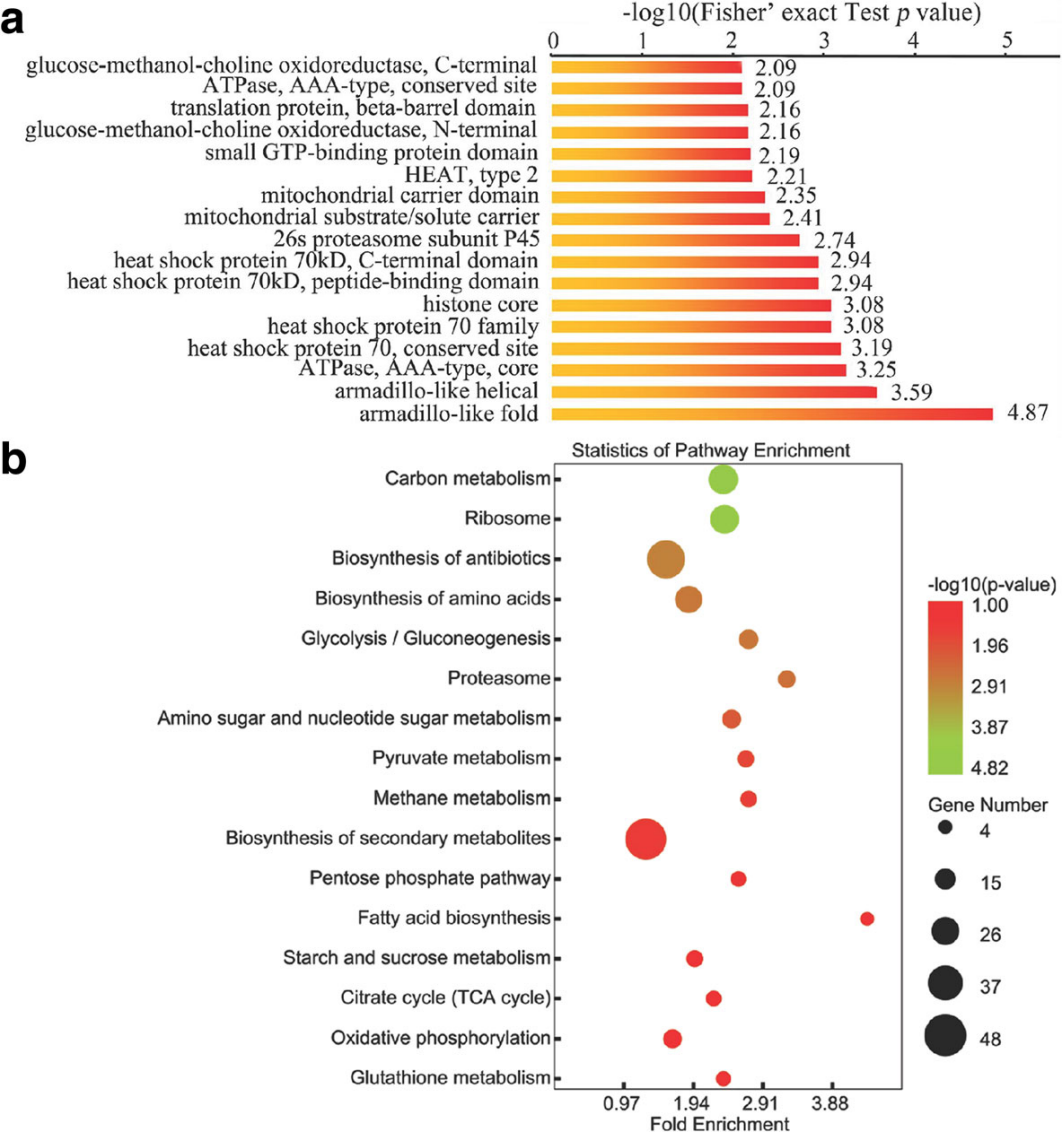
Enrichment analysis of the acetylated proteins in *F. graminearum*. **a** Domain-based enrichment analysis of the acetylated proteins. **b** KEGG pathway-based enrichment analysis of the acetylated proteins. The InterPro domain database and KEGG database were used to annotate domain and pathway of the acetylated proteins, respectively

To better understand the characteristics of the acetylated proteins in *F. graminearum*, we conducted functional enrichment of GO (biological process, molecular function, and cellular component categories), protein domain and KEGG pathway analyses (Additional file 3: Table S2, Additional file 4: Table S3 and Additional file 5: Table S4). As shown in Additional file 1: Figure S2, lysine acetylation in *F. graminearum* was involved in a broad range of functional processes. In the biological process category, most of the lysine-acetylated proteins were involved in the metabolic and catabolic processes of substances such as carbohydrate, hexose and glucose (Additional file 1: Figure S2 and Additional file 3: Table S2). Based on molecular function analysis, proteins related to constituent of ribosome, ribonucleotide, nucleotide and ion binding were significantly enriched (Additional file 1: Figure S2 and Additional file 4: Table S3). This pattern suggests that acetylation plays a vital role in saccharides compounds metabolism, protein metabolism and gene transcription regulation. Consistent with these results, most of the modified proteins were distributed in cytosol, chromatin and DNA complex (Additional file 1: Figure S2 and Additional file 5: Table S4). The domain enrichment analysis showed that proteins with armadillo-type and armadillo-like domains have a higher tendency to be acetylated (Fig. 3a, Additional file 6: Table S5). Armadillo-domain containing proteins are abundant eukaryotic proteins which mediate protein-protein interactions in diverse cellular processes including signaling, cytoskeletal regulation, transcriptional activation, nuclear transport, and cell junction assembly [39, 40]. The presence of abundant armadillo-like domains enriched in *F. graminearum* is in agreement with the involvement of lysine acetylation in diverse cellular processes.

To gain further insights into the processes regulated by the acetylation, we mapped the acetylated proteins to KEGG pathways (Fig. 3b, Additional file 7: Table S6). The results showed that proteins were enriched in several conserved pathways such as carbon metabolism and ribosomes. Furthermore, the enrichment of pathways in *F. graminearum* termed biosynthesis of antibiotics, biosynthesis of secondary metabolites and sugar metabolism (Fig. 3b, Additional file 7: Table S6) suggests that lysine acetylation may contribute to its pathogenesis and response to stress conditions.

### Acetylated proteins involved in virulence and DON biosynthesis

Functional enrichment analyses of identified acetylated proteins indicated that lysine acetylation may play an important role in *F. graminearum* pathogenesis. Consistent with this hypothesis, ten proteins involved in the virulence or DON biosynthesis of *F. graminearum* were found to be acetylated, including 4 transcription factors, 4 protein kinases and 2 phosphatases (Table 1) [41–43]. The MS/MS spectra and peak assignments for the acetylated peptides of these proteins were presented in Additional file 1: Figure S3. *F. graminearum* produces various secondary metabolites including the mycotoxin DON, which has been identified as a virulence factor [44–46]. Interestingly, Gpmk1, homologous to the *Saccharomyces cerevisiae* mating/filamentation MAPKs Fus3/Kss1, was identified as an acetylated protein in *F. graminearum*. The *S. cerevisiae* mating pheromone response pathway is a well-characterized MAPK signal transduction pathway [47, 48], in which Fus3/Kss1 is the core element. In *F. graminearum*, Gpmk1 (the orthologue of Fus3/Kss1) is involved in vegetative differentiation and virulence [49, 50]. In addition, Gpmk1 is involved in a variety of virulence-related functions in *F. graminearum* [51]. The results of inoculation with the gene deletion mutant Δ*Fg5894* and Δ*Fg9532*, respectively showed that the virulence of the two mutants on wheat was significantly reduced. And further, DON biosynthesis defect was detected in the two mutants, in particular, there was no DON was detected in Δ*Fg9532* [43]. It was found that most of the acetylation sites are located in the conserved/important domains of these ten proteins. For example, the acetylated lysine residue, K355, is within the catalytic domain of Kin4, and K413 is distributed in the acyl-CoA dehydrogenase domain of GzMyb016. Inactivation of *MoAcat1* and *MoAcat2* that encoding acetoacetyl-CoA acetyltransferases leads to defect in virulence of *Magnaporthe oryzae* [52]. In addition, increasing evidence showed that lysine acetylation functions in regulating the pathogenesis in *Mycobacterium tuberculosis* [53] and parvorius [54]. Interestingly, some virulence related proteins were identified to be acetylated in *P. sojae* [17] and *B. cinerea* [18] by acetylome analysis. Overall, these findings suggest that acetylation might play a role in virulence in *F. graminearum*.

**Table 1:** Acetylated proteins involved in virulence and DON biosynthesis of *F. graminearum*

Protein	GeneBank accession no.	Annotation	Positions	Function	Reference
GzBrom002	XP_011324943.1	Transcription Factor	461,465,469	Virulence, DON	[41]
GzC2H045	XP_011328231.1	Transcription Factor	651,997	Virulence	[41]
GzMyb016	XP_011328047.1	Transcription Factor	413	DON	[41]
GzCCHC011	XP_011327910.1	Transcription Factor	31	Virulence, DON	[41]
Gpmk1	XP_011325047.1	Mitogen-Activated Protein Kinases	335	Virulence, DON	[42, 49–51]
Kin4	XP_011316402.1	Serine/Threonine Kinases	355	Virulence	[42]
Sty1	XP_011328096.1	Mitogen-Activated Protein kinase	130	Virulence	[42]
Fg06878	XP_011326538.1	Calcium/calmodulin-dependent protein kinase	113	DON	[42]
Fg05894	XP_011324500.1	Serine/threonine protein phosphatase 2A	338	Virulence, DON	[43]
Fg09532	XP_011328185.1	Phosphatases	269	Virulence, DON	[43]

### Protein-protein interaction network of acetylated proteins

**Fig. 4:**
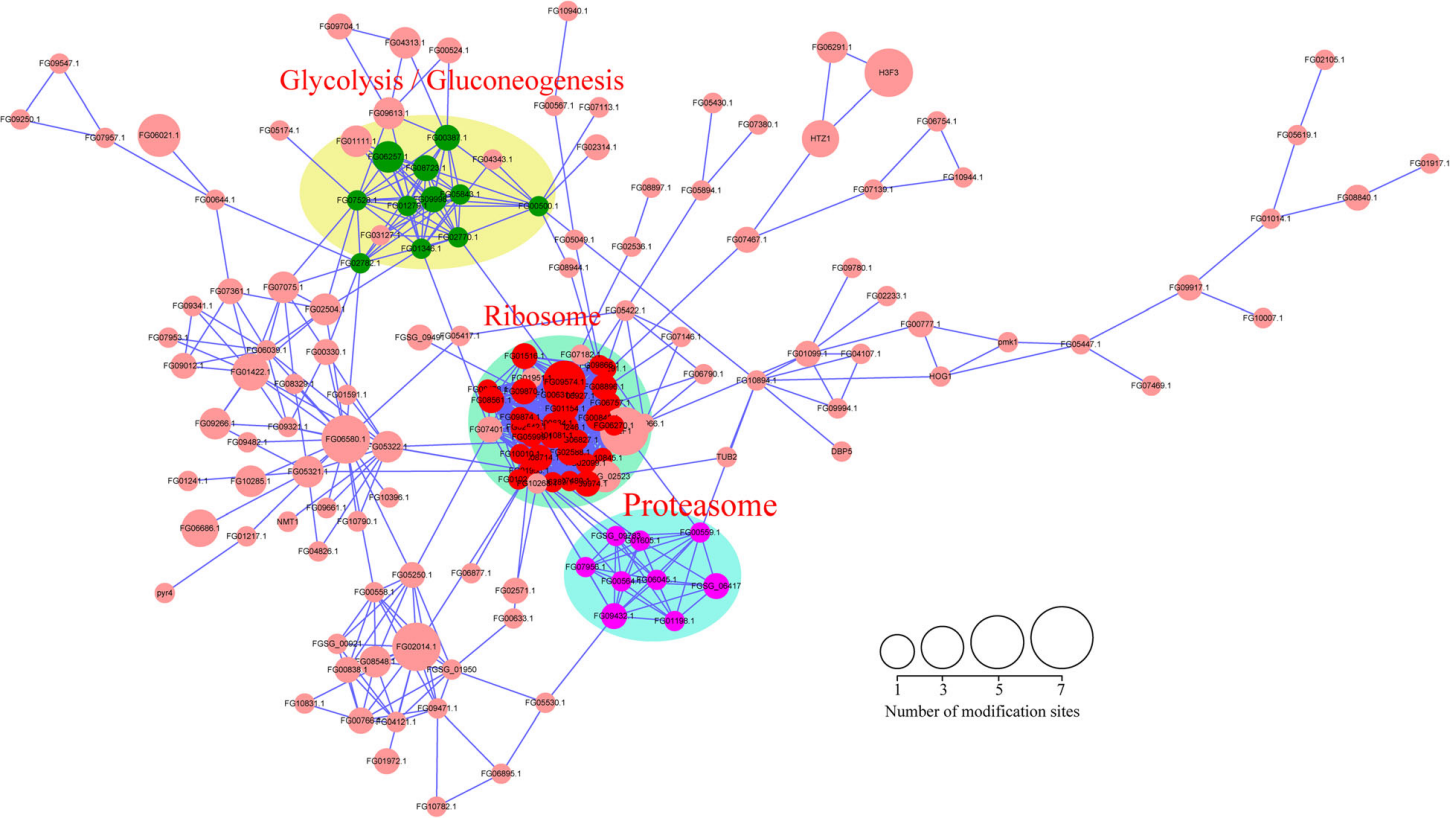
Interaction networks of the acetylated proteins in *F. graminearum*. A total of 196 acetylated proteins in *F. graminearum* were mapped to the protein interaction networks using STRING database. The clusters of glycolysis/gluconeogenesis, ribosome and proteasome associated proteins were shown in *green*, *red* and *purple*, respectively

In order to investigate cellular processes involving acetylated proteins in *F. graminearum*, the protein interaction network was established (Fig. 4). The results showed that a total of 196 acetylated proteins were mapped to the protein interaction database (Fig. 4, Additional file 8: Table S7 and Additional file 9: Table S8), which presents a global view of how acetylated proteins perform diverse types of functions in *F. graminearum*. According to the algorithm of Cytoscape software, three highly interconnected clusters of acetylated proteins were retrieved including proteins associated with glycolysis, ribosome and proteasome (Fig. 4, Additional file 1: Figures S4–S6, Additional file 8: Table S7 and Additional file 9: Table S8). The subnetwork graphs of these three pathways revealed that they all comprise a dense protein interaction network (Additional file 1: Figure S4–S6). Based on these data, we propose that acetylation is a crucial PTM of proteins in *F. graminearum* that contribute to cooperation and coordination among various pathways.

## Conclusions

In this study, we identified 577 lysine acetylation sites in 364 proteins in *F. graminearum* using a high-resolution LC-MS/MS proteomic method. Our results showed that acetylated proteins, which are localized to multiple compartments, are involved in almost all cellular processes. Our findings reinforce the notion that lysine acetylation plays a crucial regulatory role in diverse aspects of cellular processes in *F. graminearum*. This study widens the range of physiological processes regulated by lysine acetylation and provides a rich resource for exploring the functions of lysine acetylation in filamentous fungi.

## Additional files

Additional file 1: Figure S1. Proteome-wide identification of lysine acetylation sites in *F. graminearum*. A, Mass error distribution of all identified peptides. B, Peptide length distribution. **Figure S2.** GO-based enrichment analysis of acetylated proteins. **Figure S3.** The MS/MS spectra and peak assignments for the acetylated peptides involved in pathogenesis of *F. graminearum*. **Figure S4.** Interaction network of acetylated proteins associated with ribosome. **Figure S5.** Interaction network of acetylated proteins associated with glycolysis/gluconeogenesis. **Figure S6.** Interaction network of acetylated proteins associated with proteasome. (DOCX 2130 kb)
Additional file 2: Table S1. The identified acetylated sites in *F.graminrarum*. (XLS 401 kb)
Additional file 3: Table S2. A GO-based enrichment analysis of the acetylated proteins in terms of biological process. (XLS 94 kb)
Additional file 4: Table S3. A GO-based enrichment analysis of the acetylated proteins in terms of molecular function. (XLS 74 kb)
Additional file 5: Table S4. A GO-based enrichment analysis of the acetylated proteins in terms of cellular component. (XLS 38 kb)
Additional file 6: Table S5. Enrichment of protein domain analysis. (XLS 44 kb)
Additional file 7: Table S6. KEGG pathway enrichment analysis. (XLS 25 kb)
Additional file 8: Table S7. Protein-protein interaction network of indentified acetylated proteins. (XLSX 57 kb)
Additional file 9: Table S8. Data analysis of acetylated proteins in protein-protein interaction network. (XLSX 96 kb)
