# Supplementary material for: Systematic analysis of the lysine acetylome in Fusarium graminearum
Source: BMC Genomics. 2016 Dec 13;17:1019. doi: 10.1186/s12864-016-3361-3 (PMC5153868; doi:10.1186/s12864-016-3361-3)
Supplement: Additional file 1: Figure S1. — Proteome-wide identification of lysine acetylation sites in F. graminearum. A, Mass error distribution of all identified peptides. B, Peptide length distribution. Figure S2. GO-based enrichment analysis of acetylated proteins. Figure S3. The MS/MS spectra and peak assignments for the acetylated peptides involved in pathogenesis of F. graminearum. Figure S4. Interaction network of acetylated proteins associated with ribosome. Figure S5. Interaction network of acetylated proteins associated with glycolysis/gluconeogenesis. Figure S6. Interaction network of acetylated proteins associated with proteasome. (DOCX 2130 kb) [file 12864_2016_3361_MOESM1_ESM.docx]

**Systematic analysis of the lysine acetylome in *Fusarium graminearum***

Shanyue Zhou^a^, Qianqian Yang ^a^, Changfa Yin^b^, Lin Liu^c^, Wenxing Liang^a,*^

^a^College of Agronomy and plant Protection, The Key Lab of Integrated Crop Pests Management of Shandong Province, Qingdao Agricultural University, Qingdao 266109, China.

^b^College of plant Protection, China Agricultural University, Beijing 100193, China.

^c^College of Life Sciences, Shandong Province Key Laboratory of Applied Mycology, Qingdao Agricultural University, Qingdao 266109, China.

**^*^Corresponding author:** College of Agronomy and Plant Protection, Qingdao Agricultural University, No. 700 Changcheng Road, Chengyang, Qingdao, Shandong 266109, China; wliang1@qau.edu.cn

**Fig. S1.** Proteome-wide identification of lysine acetylation sites in *F. graminearum*. A, Mass error distribution of all identified peptides. B, Peptide length distribution.


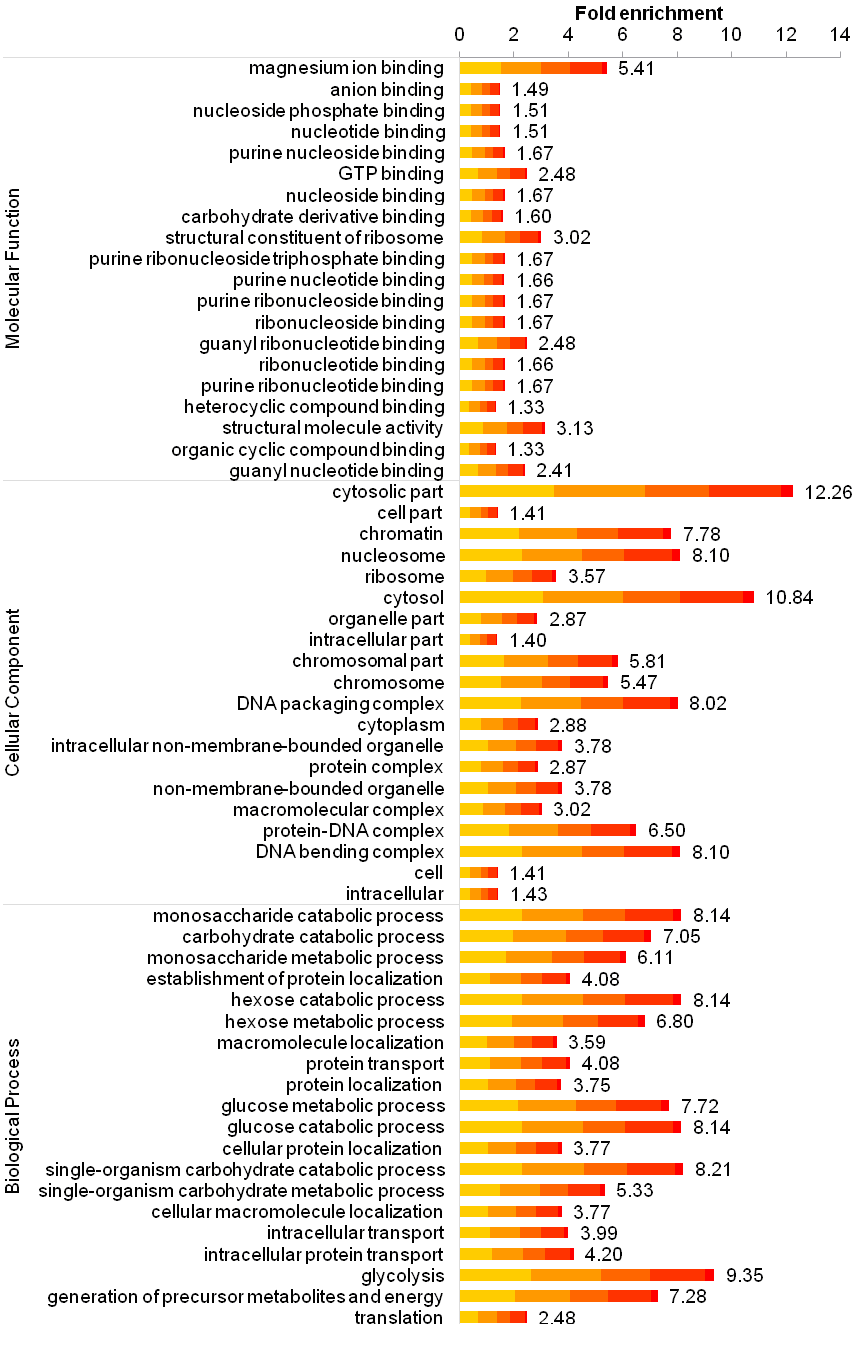


**Fig. S2.** GO-based enrichment analysis of acetylated proteins.


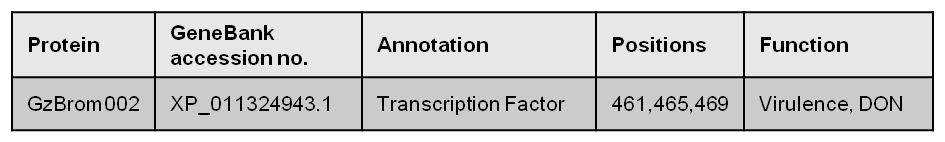

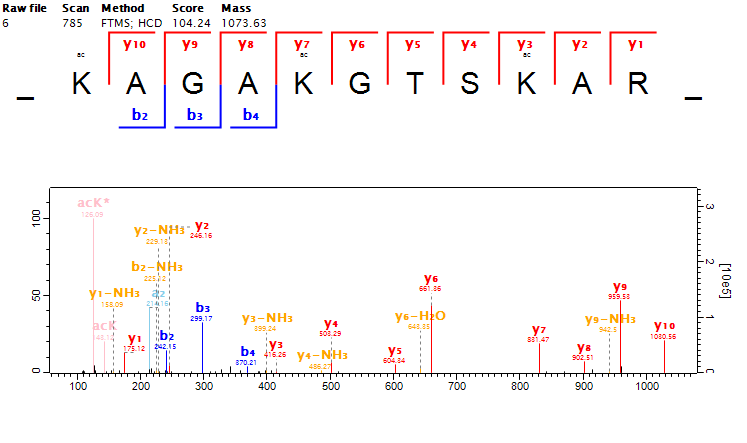

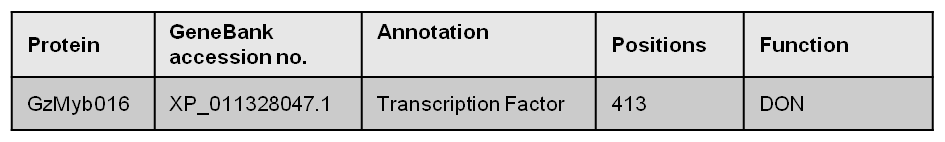

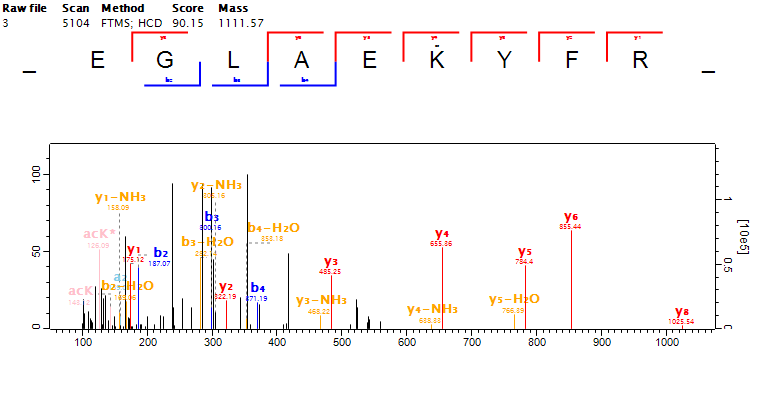


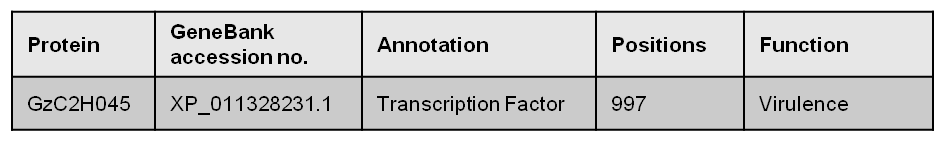


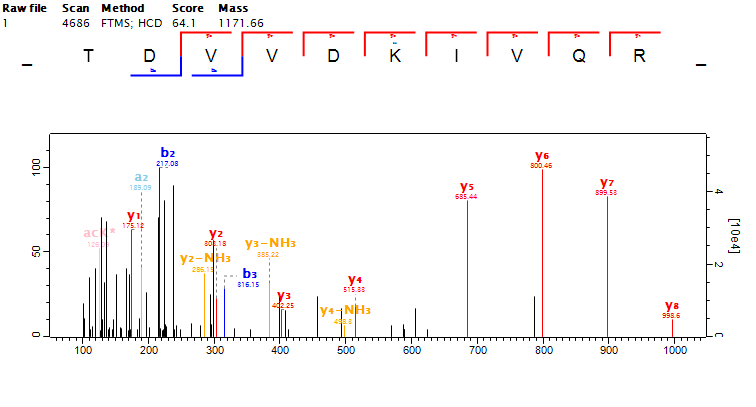

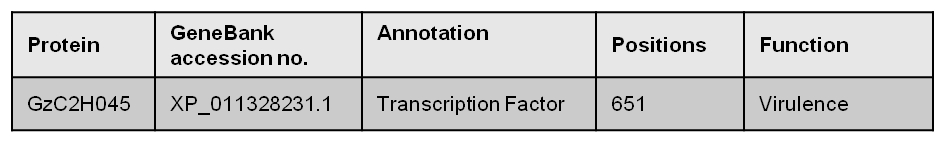

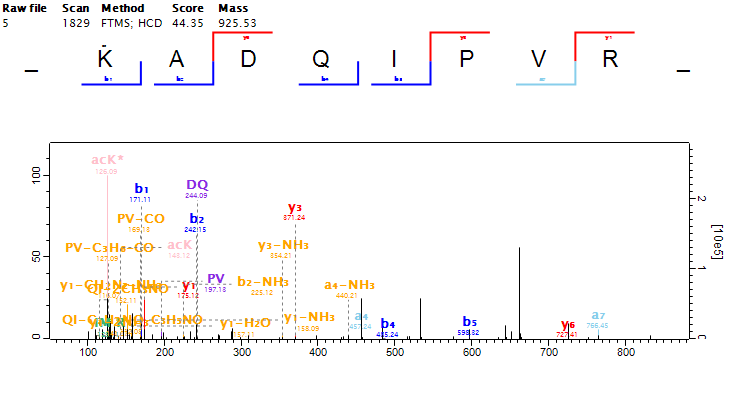


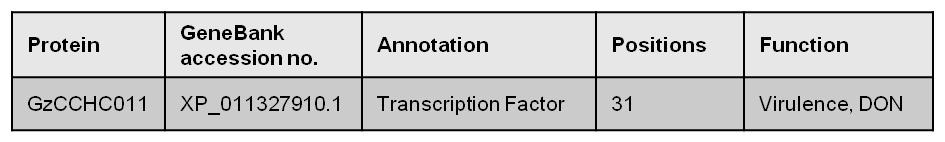

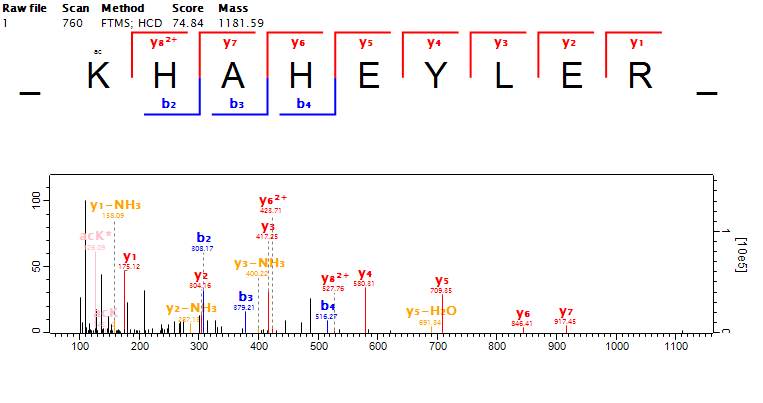

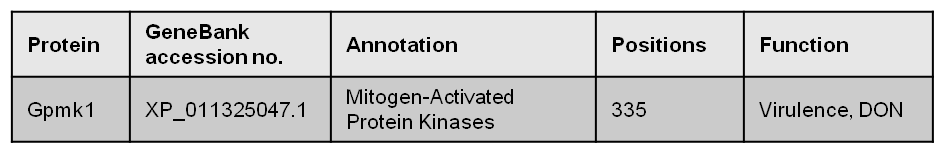

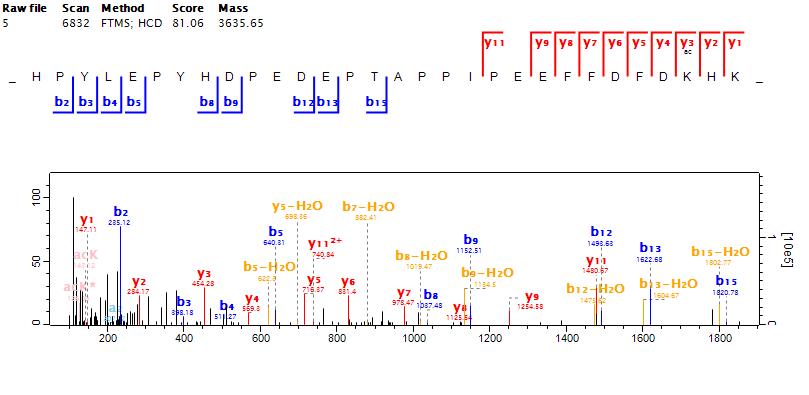


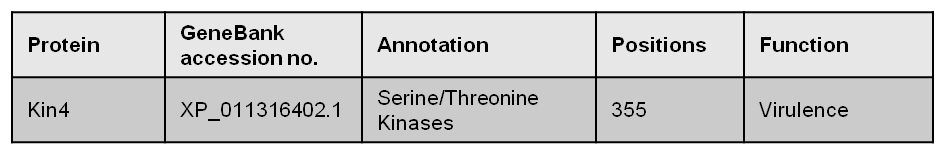

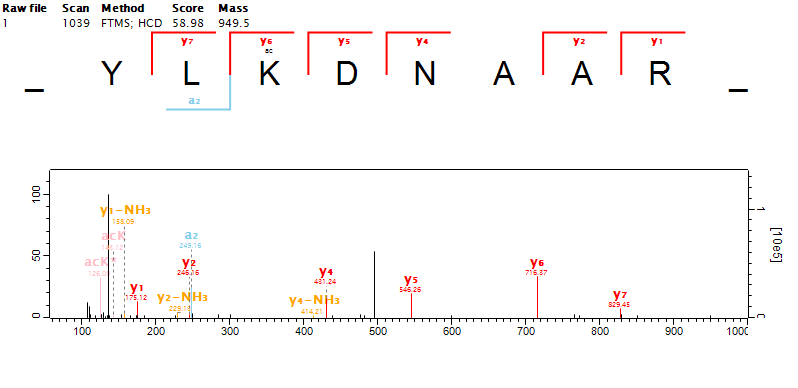

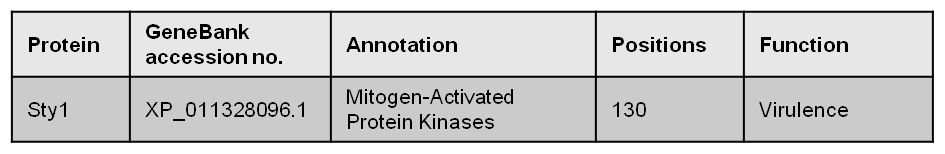

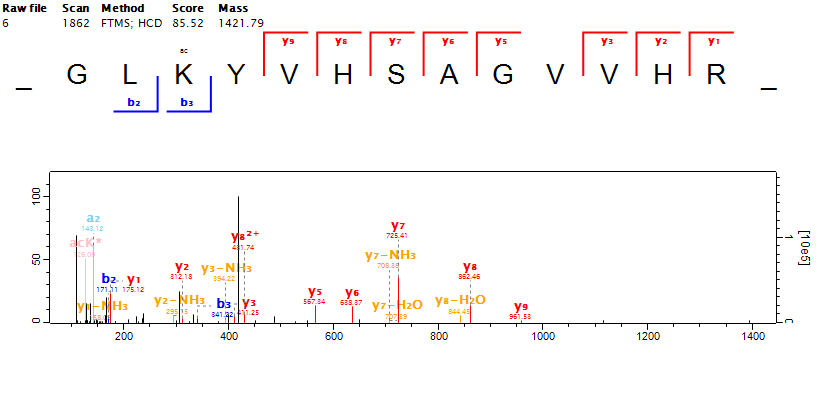


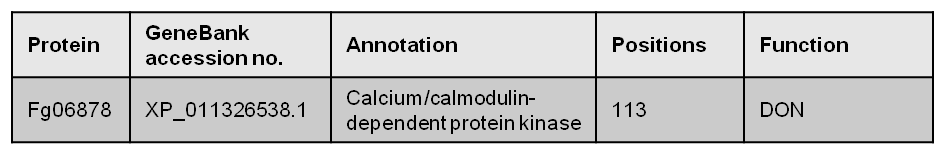

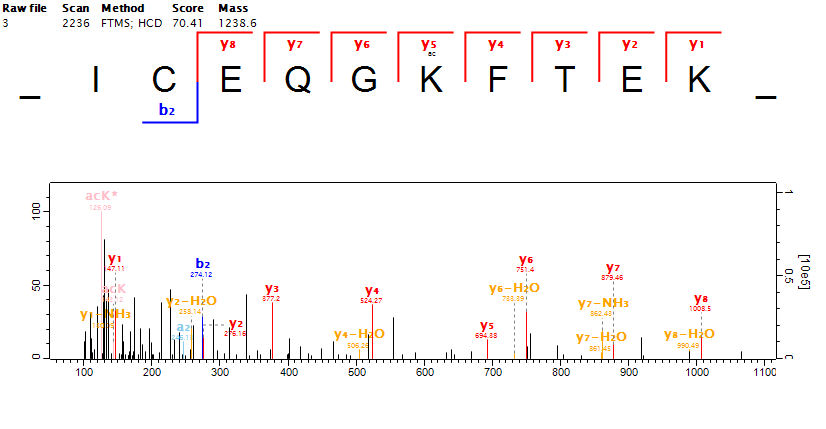

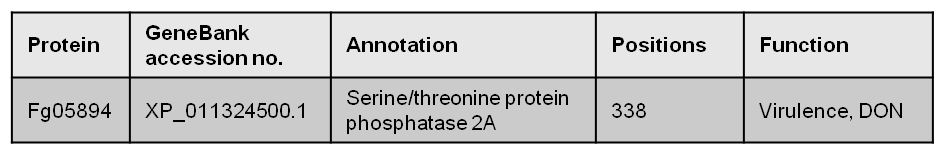

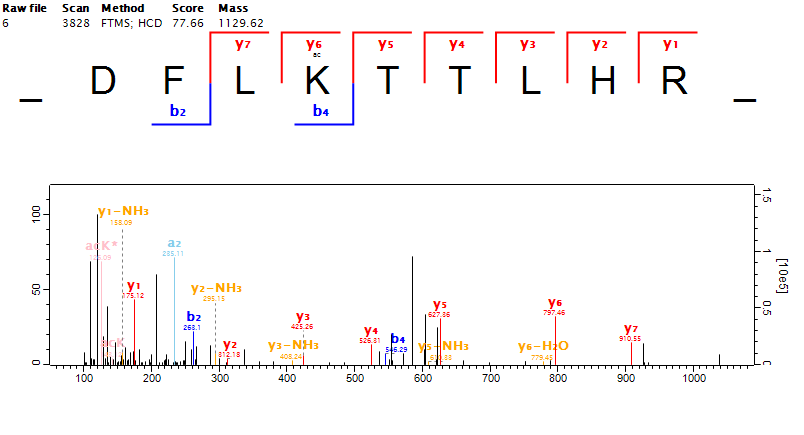


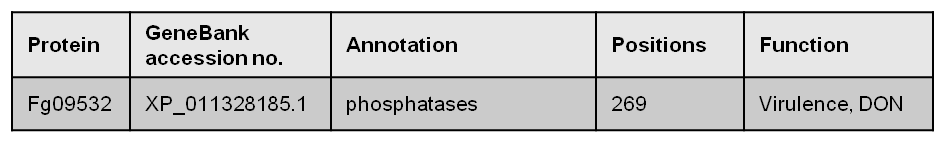

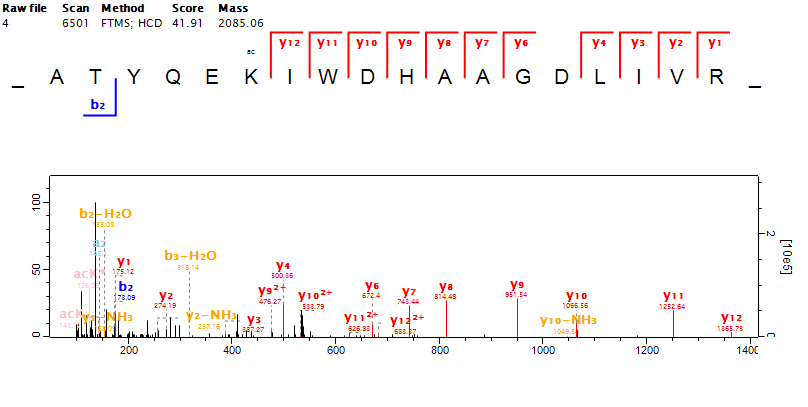


**Fig. S3.** The MS/MS spectra and peak assignments for the acetylated peptides involved in pathogenesis of *F. graminearum.*

.


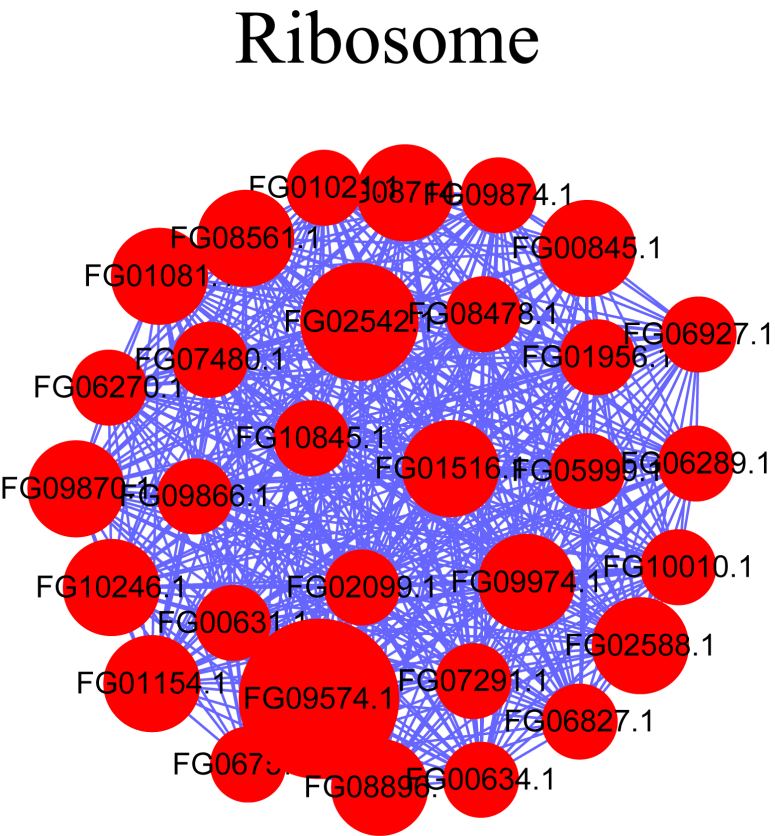


**Fig. S4.** Interaction network of acetylated proteins associated with ribosome.


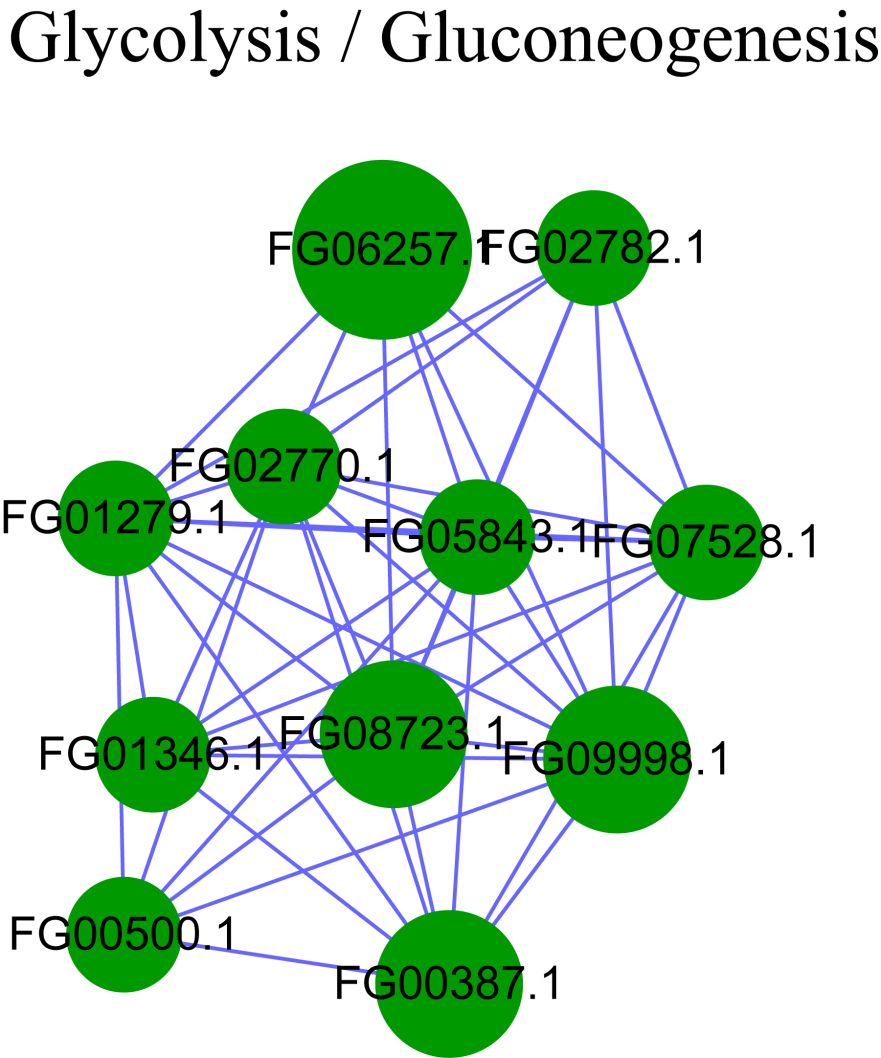


**Fig. S5.** Interaction network of acetylated proteins associated with glycolysis/gluconeogenesis.


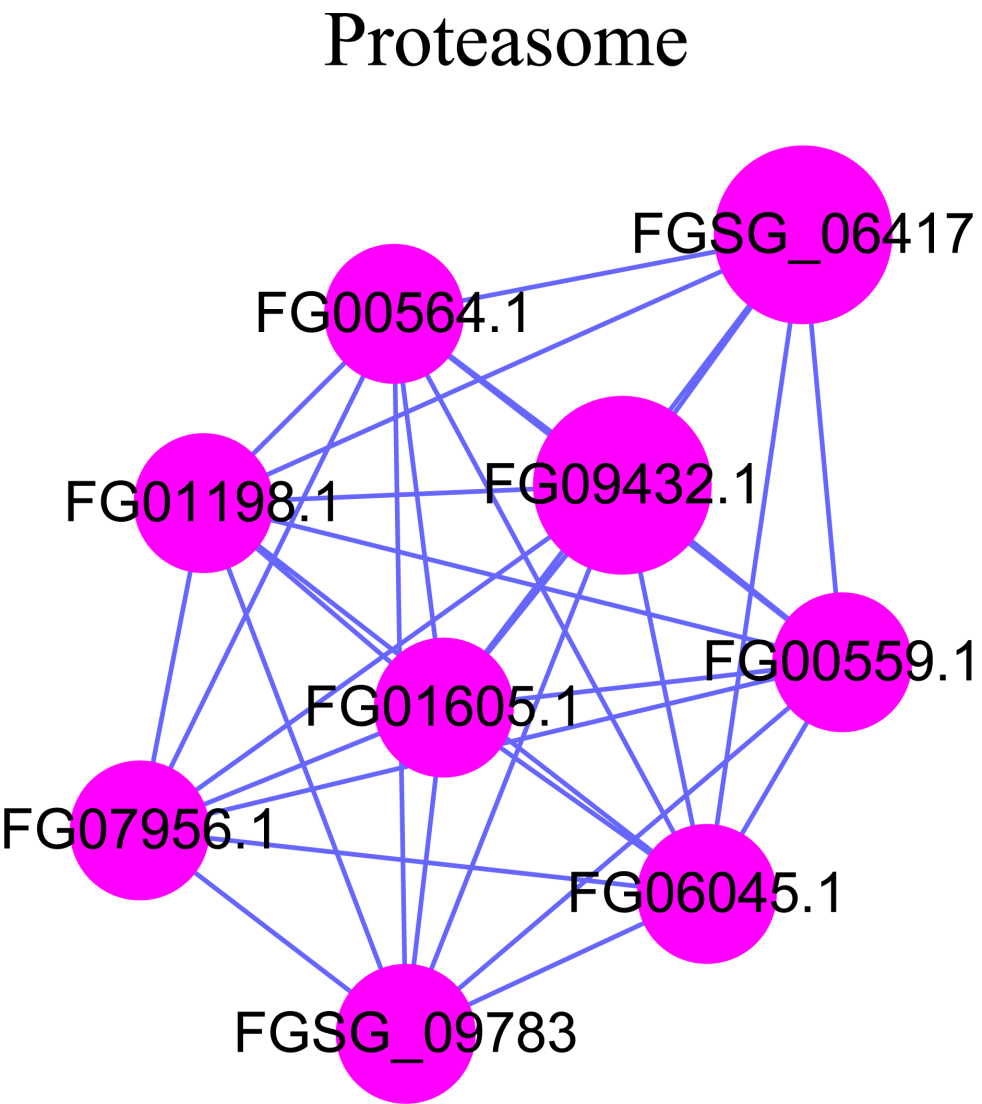


**Fig. S6.** Interaction network of acetylated proteins associated with proteasome.
